# Reverse rolling-mat type lymph node dissection is the key step to solve the operative difficulties in hand-assisted laparoscopic D2 radical gastrectomy

**DOI:** 10.1186/s12893-021-01460-4

**Published:** 2022-01-08

**Authors:** Peng Shu, Long Cheng, Chuan Xie, Jun Zhou, Qianjun Yu, Xin Dai, Siping Chen, Qiang Wang, Yongkuan Cao, Tao Wang

**Affiliations:** 1 Department of General Surgery, The General Hospital of Western Theater Command, Chengdu, 610083 People’s Republic of China; 2 Department of Hepatobiliary Surgery, School of Clinical Medicine, Southwest Medical University, Luzhou, Sichuan 646000 People’s Republic of China

**Keywords:** Gastric cancer, Hand-assisted laparoscopic, Reverse rolling-mat type lymph node dissection, Cabbage type lymph node dissection, Surgical outcomes

## Abstract

**Background:**

We have improved and named a new reverse rolling-mat type lymph node dissection, which effectively solves the dilemma faced by the traditional lymph node dissection in hand-assisted laparoscopic D2 radical gastrectomy through the optimization of the surgical procedure. However, the relevant clinical data are still scarce. The study aims to compare the clinical effects of two surgical procedure and explore the safety and feasibility of “reverse procedure”.

**Study design:**

The clinicopathological data of 195 patients who underwent hand-assisted D2 radical total gastrectomy (HALTG) in our hospital from January 2011 to September 2017 were collected. A retrospective case–control study was used to compare the clinical outcomes of the two patterns of lymph node dissection. Among them, 89 patients underwent “cabbage type” lymph node dissection and 106 patients underwent the “reverse procedure” lymph node dissection.

**Results:**

There were no significant differences between the two groups of patients in terms of gender, age, tumor location, incision length, postoperative hospitalization duration, pathological classification, recent complications, long-term recurrence and metastasis. The operation time of “cabbage type” group was shorter than that of “reverse procedure” group (178.35 ± 31.52 min vs 191.25 ± 32.77 min; *P* = 0.006). While, in the “reverse procedure” group, intraoperative blood loss was less (249.4 ± 143.12 vs 213.58 ± 101.43; *P* = 0.049), and there were more numbers of lymph nodes dissected (18.04 ± 7.00 vs 32.25 ± 14.23; *P* < 0.001).

**Conclusion:**

The pattern of reverse rolling-mat type lymph node dissection in HALTG perform well in terms of safety and feasibility.

## Introduction

Gastric cancer is one of the most malignant tumor types worldwide with high morbidity and mortality [1–3]. Surgery is the best treatment for patients with resectable gastric cancer, and D2 lymphadenectomy is recommended as the standard surgical approach for patients with curable gastric cancer [4]. Studies have shown that the HALG can achieve the same results of operations as traditional open surgery, but also has a minimally invasive effect comparable to laparoscopic-assisted radical gastrectomy (LAG). In the western population, the body mass index (BMI) is generally higher than that in the Asian population, and according to our experience, it is still difficult to operate in robot-assisted and laparoscopic-assisted radical gastrectomy [5]. However, with the advantages of hand tactile feedback and intraoperative resistance to traction of tissue, HALG can reduce the complexity of surgical operations and has higher surgical safety, especially in those with obesity. Therefore, it is not a simple copy of laparoscopic surgery, but a new operative procedure [6–8].

Radical resection and appropriate lymph node dissection are necessary to improve the outcome of gastric cancer surgery [9–12]. Prior to this, it mainly depended on the “traditional lymph node dissection procedure” which started from two opposite directions and finally converged at celiac trunk for the HALTG; we named this procedure “cabbage type lymph node dissection” due to the pattern of lymph node dissection seems similar to a cabbage, which gradually folds from outside to inside. However, the traditional pattern of lymph node dissection (cabbage type lymph node dissection) still faces many challenges, such as obesity, history of upper abdominal surgery, and huge gastric tumors and so on, which limit the development of the HALG.

In recent years, with increasing clinical practice and the thinking on the improvement of surgery, we have improved and named a new lymph node dissection pattern based on the traditional cabbage procedure of the HALG-*The reverse rolling-mat type lymph node dissection* [13]. Continuous clinical practice has proved that it is more practical and safer after optimizing the surgical procedure. At present, there is still a lack of comparison of the surgical outcome of these two different lymph node dissection methods in total gastrectomy. Therefore, this article aims to compare the short-term and long-term surgical outcomes of patients who under the HALTG between the reverse rolling-mat type lymph node dissection and cabbage type lymph node dissection, and explore the safety and feasibility using of reverse procedure in HALTG.

## Materials and methods

Clinicopathological data of 195 patients with gastric cancer were collected from the General Hospital of Western Theater Command from January 2011 to September 2017 which through gastroscopy histopathological diagnosis, CT examination and other examinations to assess the resectable lesions, and received the HALTG. Inclusion criteria were as follows: histologically confirmed gastric malignant tumor; no evidence of distant metastasis by means of abdominal computed tomography (CT) and/or abdominal ultrasound and posteroanteriorchest radiograph. Exclusion criteria were as follows: intraoperative evidence of peritoneal disseminated or distant metastasis; incomplete of pathological data. The all patients were divided into the conventional cabbage type group and the reverse procedure group according to two types of sweeping; Among them, 89 patients received cabbage type technical approach for lymph node dissection (cabbage type: centered on the celiac trunk, and lymph nodes were dissected from both sides to the middle) and 106 cases of patients received reverse rolling-mat type lymph node dissection (reverse procedure: sweeping lymph nodes from left to right). The patients were followed up through telephone, outpatient and inpatient review after the operation. This study was discussed and approved by the Ethics Committee of the General Hospital of the Western Theater Command, and was approved by the patients and their families.

### Surgical procedures

All patients in the group chose hand-assisted laparoscopic surgery voluntarily and signed an informed consent form for the operation. D2 lymphadenectomy was performed according to the Japanese Classification of Gastric Carcinoma (13th edition). After general anesthesia, the patients were positioned supine on the operating table, and the “three-step procedure” was used to implement HALTG [14, 15]. The brief procedure is as follows: (1) First step: A 6 to 8 cm exploratory incision below the median xiphoid process of the upper abdomen was made into the abdominal cavity and then a LapDisc hand-assisted device was placed into the opened incision providing surgical field. After completing the abdominal cavity exploration, perform partial lymph node dissection under direct vision if available. (2) Second step: partial regional lymph node was dissected under the hand-assisted laparoscopic. (3) Third step: The tumor and stomach are removed, and the digestive tract reconstruction was performed through the incision; Completing partial lymph node dissection under direct vision when required, such as lymph node dissection of No. 6 group below the pylorus, etc. At the end of the procedure, a drainage tube was placed in the peritoneal cavity before skin closure.

### Lymph node dissection

**Fig. 1:**
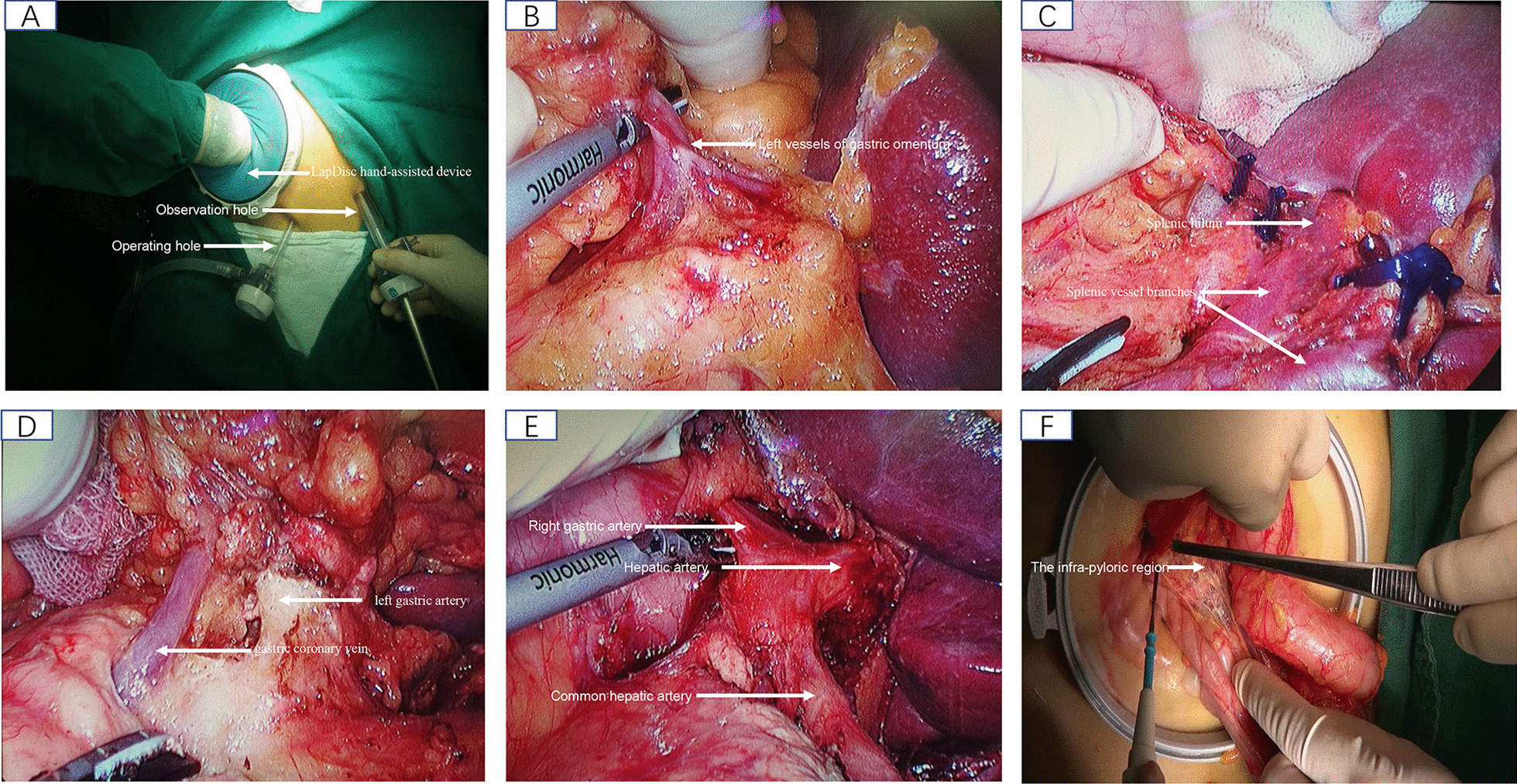
Reverse rolling-mat type lymph node dissection for total gastrectomy (**A**–**F**); **A** shows that the establishment of surgical operating system; **B** shows that the NO. 4sb lymph nodes were dissected; **C** shows that NO. 10 and NO. 11d lymph nodes were dissected; **D** shows that NO. 11 p,9,7 and 8a lymph nodes were dissected; **E** shows that the NO. 8a, 12a and 5 lymph nodes were dissected; **F** shows that the NO. 6 lymph nodes were dissected under direct vision via auxiliary incision

1. *Cabbage type*: firstly, separate the greater omentum and the anterior lobe of the Transverse Colon Mesentery, and clean sequentially groups of No. 6, No. 5, No. 12a/No. 8a and No. 9 Lymph nodes under direct vision through a small 7 cm exploratory incision in the upper abdomen. Next, dissect sequentially groups of 4sb, No. 10, No. 11d and No. 2 and then lymph node groups of No. 11p, No. 7, No. 1 and No. 3 were removed by hand-assisted laparoscopy after establishing the pneumoperitoneum. Converge with the area of surgical dissection of direct vision around the celiac trunk eventually [16–18].
2. *Reverse sweeping type*: the procedure is also divided into two sections: (1) after completing the abdominal cavity exploration through a small exploration incision, we establish pneumoperitoneum, and perform sequential lymph nodes follow the order by the groups of No. 4sb, No. 10, No. 2 and No. 11d (left zone of the gastric bare area). Then, we remove the lymph node groups of No. 11p, No. 9, No. 1 and No. 3 (right zone of the gastric bare area). (2) lymph node groups of No. 8, No. 5, No. 12a, and No. 6 was dissected under direct vision after the excised stomach and omentum were removed from the abdominal cavity. Of course, this part is flexible, because the groups of No. 8, No. 5, No. 12a is sometimes more suitable be performed under laparoscopy. However, in the vast majority of cases, the lymph nodes in the No.6 group need to be performed under direct vision. The intraoperative photos showed the surgical procedures of reverse lymph node dissection, as detailed in Fig. 1.

After the laparoscopic operation, Roux-en-Y or Braun's anastomosis were mainly chosed to reconstruct the digestive tract according to the inpatient's condition and results of the intraoperative exploration. Toward the end of the surgery, a drainage tube was placed routinely and the wound was closed after cleaning up the abdominal cavity. The postoperative pathological specimens were sent to the pathology department. Eventually, the pathologist and the surgeon jointly found the number of lymph nodes that were dissected, and the pathologist determined the final diagnosis.

### Observation indicators

The evaluation of surgical safety and feasibility refers to the Brenkman's study [19]. The following parameters were recorded: operation time, estimated blood loss, number of dissected lymph nodes, intraoperative complications, postoperative complications, cases of recurrence or metastasis, deaths and so on.

### Statistics

The relevant indicators before and after surgery of enrolled patients were collected, and data were analyzed using IBM SPSS statistics (version 25.0). The Categorical data were expressed in numerical form, and statistical analysis was performed by chi-square test or Fisher's exact test. Metric data were described using means, and standard deviations (SDs). Statistical analysis was performed with a T-test for Count data. A Mann–Whitney test was applied for the distribution of the statistics were skewed and not symmetric. P value < 0.05 was considered to have statistical significance.

## Results

A total of 204 patients were planned to undergo total gastrectomy, of which 9 patients were excluded for the following reasons: 6 cases underwent palliative resection due to extensive abdominal metastasis was found through intraoperative exploration; 1 case was performed combined pancreas and spleen resection due mainly to its late diagnosis; 2 cases were excluded due to incomplete intraoperative and postoperative data. 195 patients met the preoperative evaluation criteria and were performed in HALTG. Among them, 89 cases used the “cabbage type” method for lymph node dissection, and 106 cases used the “reverse procedure” for lymph node dissection. The research results are reported as follows.

### Comparison and analysis of general data of the two groups of patients

There were 195 cases in the cabbage groups and the reverse groups. There was no statistical difference between the two groups in terms of age, gender, tumor size, tumour sites, and postoperative pathological type. We have tended to use the reverse rolling-mat type lymph node dissection for the patients who had greater weight according to clinical experience. Therefore, the body mass index of the “reverse procedure” group is higher than that of the “cabbage type” group. Details are shown in Table 1.

**Table 1:** Comparison and analysis of general data of the two groups

	Cabbage type (n = 89)	Reverse procedure type (n = 106)	*P* (value)
Age (years)	60.26 ± 9.50*	62.45 ± 9.27*	0.105^b^
Sex (%)			0.126^a^
Male	63 (70.8)	85 (80.2)	
Female	26 (29.2)	21 (19.8)	
BMI (kg/m^2^)	20.53 ± 3.49*	22.40 ± 2.92*	< 0.001^b^
Tumour size (cm)	5.12 ± 2.18*	5.41 ± 2.97*	0.448^b^
Tumour sites (%)			0.933^a^
Cardia	31 (34.8)	39 (36.8)	
Gastric fundus	12 (13.5)	12 (11.3)	
Gastric body	36 (40.4)	45 (42.5)	
Gastric antrum	10 (11.2)	10 (9.4)	
Pathological differentiation (%)			
High differentiation	1 (1.1)	1 (0.9)	> 0.99^a^
Middle differentiation	31 (34.8)	36 (34.0)	0.90^a^
Middle-low differentiation	15 (16.9)	20 (18.9)	0.715^a^
Low differentiation	42 (47.2)	49 (46.2)	0.893^a^

^*^Values are mean ± standard deviation

^a^Chi square test or Fisher’s exact test except

^b^Student’s t test

### Analysis of relevant data before and after the operation of the two groups

There was no significant difference between the two groups in terms of incision length, postoperative hospitalization duration. The comparison of the operation time between the “cabbage type” group and the “reverse procedure” group showed that the latter had a longer operative time (178.35 ± 31.52 min vs 191.25 ± 32.77 min; *P* = 0.006), but it performed more satisfactorily in terms of reduced operative blood loss (249.4 ± 143.12 ml vs 213.58 ± 101.43 ml; *P* = 0.049). Another a result was unexpected that more numbers of harvested lymph nodes in the “reverse procedure” group than the “cabbage type” group significantly (18.04 ± 7.00 vs 32.25 ± 14.23; *P* < 0.001), and the comparison between the two groups was statistically significant. In our study, the “cabbage type” group used more Braun's anastomosis for gastrointestinal reconstruction during the operation, while the “reverse procedure” group used more Roux-en-Y anastomosis methods. Choice of two methods of reconstruction of digestive tract after total gastrectomy mainly based on the patient's condition and results of the intraoperative exploration. But this does not affect our intraoperative lymph node dissection. Details are shown in Table 2.

**Table 2:** Analysis of relevant data before and after the operation of the two groups

	Cabbage type (n = 89)	Reverse procedure type (n = 106)	*P* (value)
Operative time (min)	178.35 ± 31.52*	191.25 ± 32.77*	0.006^b^
Postoperative			
Hospital stay (days)	10 (8, 11)	9 (9, 11)	0.85^c^
Blood loss (ml)	249.4 ± 143.12*	213.58 ± 101.43*	0.049^b^
Incision length (cm)	6.94 ± 0.24*	7.08 ± 0.52*	0.014^b^
Mean number of retrieved lymph nodes	18.04 ± 7.00*	32.25 ± 14.23*	< 0.001^b^
Methods of digestive tract reconstruction (%)			0.030^a^
Roux-en-Y	34 (38.2)	57 (53.8)	
Braun	55 (61.8)	49 (46.2)	

^*^Values are mean ± standard deviation

^a^Chi square test or Fisher’s exact test except

^b^Student’s t test

^c^Mann–Whitney test

### Comparison of postoperative recovery between the two groups

Our study compares the complications within 3 months and the results of recurrence, metastasis, and death within 1 year in the two groups after surgery. During the follow-up period, recurrence and/or metastasis occurred in 10 patients in the “cabbage type” group and 5 patients in the “reverse procedure” group. Within 1 year, there were 1 death case occurred between the two groups, respectively. There were no significant differences in postoperative complications, number of reoperations, recurrence, metastasis, and death between the two groups of patients, and the statistical results showed no statistical significance. Details are shown in Table 3.

**Table 3:** Comparison of postoperative short-term complications and long-term outcome between the two groups

	Cabbage type (n = 81)	Reverse procedure type (n = 95)	*P* (value)
Complications (%)	12	15	0.835^a^
Intestinal obstruction	2 (2.5)	2 (2.1)	> 0.99^a^
Ascites or peritoneal effusions	2 (2.5)	5 (5.3)	0.45^a^
Pleural effusion	5 (6.2)	6 (6.3)	0.97^a^
Intra-abdominal infection	0 (0)	1 (1.1)	> 0.99^a^
Duodenal stump leakage or Anastomotic leak	2 (2.5)	1 (1.1)	0.59^a^
Gastrointestinal bleed	1 (1.2)	0 (0)	0.46^a^
Reoperation	2 (2.5)	1 (1.1)	0.593^a^
Recurrence or metastasis	10 (12.3)	5 (5.3)	0.093^a^
Deaths	1 (1.2)	1 (1.1)	> 0.99^a^

^a^Chi square test or Fisher’s exact test except

## Discussion

Surgery is the main treatment for patients with resectable gastric cancer, and D2 lymphadenectomy is recommended as the standard surgical approach for patients with curable gastric cancer [4]. Hand-assisted laparoscopic surgery which relies on the tactile feedback, dexterity of the hand during surgical procedures and the advantages of clear and broad laparoscopic vision is widely used in various abdominal surgery operations [20–23]. In addition to that, hand-assisted laparoscopic surgery also has a shorter learning curve [24] and can achieve similar surgical results as laparoscopy-assisted gastrectomy [6]. Previous studies showed that lymph node dissection is still an inevitable difficulty for laparoscopic radical gastric cancer surgery due to the abundant blood vessels around the stomach, constriction of the visual field and the complexity of anatomy, such as the NO.6 group lymph node [9, 25]. In hand-assisted radical gastric cancer surgery, the degree of surgical operations difficulty was increased when face the problems of obese patients, omentum hypertrophy, gastric antrum tumors with huge volume, and short transverse colonic mesentery with using the “cabbage type lymph node dissection”. Especially, the performance is more difficult when dissecting the lymph nodes groups of 5, 6, 8a and 12a, because vision is blocked by a portion of the transverse colon and the stomach in the first step. The technical difficulties of lymph node dissection may restrict the development of hand-assisted laparoscopic radical gastric cancer surgery. Therefore, to find a more suitable way for hand-assisted laparoscopic radical gastrectomy for lymph node dissection is urgently needed. Over the years, we keep summarizing clinical experience and thinking about solutions during the hand-assisted laparoscopic radical gastric cancer surgery. Ultimately, we improved and named a novel pattern of lymph node dissection-the reverse rolling-mat type lymph node dissection [13], and was also known as “reverse-sheet-folding-like procedure” [18], which effectively solves the dilemma faced by the traditional lymph node dissection pattern through the optimization of the surgical procedure. In fact, many surgeons use similar lymph node dissection in complete laparoscopic or even robot-assisted gastric cancer surgery, but we elaborated and named this lymph node dissection mode for the first time.

As reported previously, the technical difficulties were associated with conventional open gastrectomy with D2 lymphadenectomy of gastric cancer in patients with high body mass index (BMI) values, since the N2 regional lymph nodes lie deep within the fatty tissues around the major abdominal vessels, and may be associated with short-term surgical outcomes and hemorrhage [26, 27]. Prior to this, it was often performed lymph node dissection using the traditional “cabbage type” in HALTG. However, it is difficult to complete the first step of the “cabbage-style” operation when facing in the obese patients, obese omental tissue, gastric antrum tumors with huge volume, and short transverse mesocolon [18]. We can't dissect smoothly under direct vision because it is difficult to drag the gastroomentum by the small operating space. Even, we have to change operation mode from laparoscopic to open surgery. On the contrary, the approach of “reverse rolling-mat type lymph node dissection” avoid effectively the difficulties of surgical operation under direct vision of the first step of using "cabbage-type lymph node dissection ". We can remove the lymph node groups of No. 8, No. 5, No. 12a, and No. 6 under direct vision because the excised stomach and omentum had been removed from the abdominal cavity before completing hand-assisted laparoscopic lymph node dissection. Of course, the lymph node groups of No. 8, No. 5, No. 12a can also be performed under laparoscopy if convenient for practical operation. Therefore, it can continue to keep the technical advantages of lymph node dissection under direct vision. In addition, it also avoids pulling and squeezing the tumor in the middle and lower stomach; at the same time, we can directly expose the celiac trunk and the common hepatic artery, which is conducive to remove groups of 8a, No. 9 lymph nodes under direct vision and the pylorus areas were more clearly revealed.

High BMI can also cause technical difficulties in laparoscopic-assisted gastric surgery for inexperienced surgeon [28, 29]. However, the difficulty of the surgical procedures is significantly reduced through combine improved reverse procedure method with the advantages of hand-assisted laparoscopy. According to our clinical experience, this novel pattern of lymph node dissection is more suitable for the patients with a larger body mass index. Therefore, in our statistical results, the average body mass index of patients in the “reverse procedure” group is greater than that of the “cabbage type” group; which may be related to that we tended to choose the “reverse procedure” for patients with heavier weight. In our study, the “reverse procedure” group had longer average surgical procedure time, but the intraoperative blood loss was significantly lower than that of the traditional “cabbage type” group. We believe that the longer operation time may be related to the fact that patients had higher average BMI in the ”reverse procedure” group; even so, the “reverse procedure” group showed it is more satisfactory in reducing intraoperative blood loss. This is the result of our optimization of the operation method, because we can more clearly reveal the anatomy through this surgical procedure. Generally speaking, obesity and the history of upper abdominal surgery will affect the lymph node dissection of D2 radical surgery for gastric cancer [30]. But, the results from our study shows unexpectedly that more lymph nodes harvested lymph nodes in the “reverse procedure” group. On the one hand, the reason is that we have optimized the surgical procedure; on the other hand, postoperative pathological specimen lymph nodes were collected by the surgeons and pathologists jointly in the “reverse procedure” group. While, in the “cabbage type” group, the lymph nodes were collected alone by the pathologist alone. Although all operations are performed by the same group of doctors, and the extent of lymph node dissection is carried out in accordance with the standard D2 radical surgery sweeping range, there is no denying that the examination of pathology specimens is a flaw in this study. Currently, we don't more research on reverse procedure in laparoscopic-assisted gastrectomy, we also believe that this optimized lymph node dissection can still be applied to the HALG well.

In addition, in our study, the follow-up rate of patients in the “cabbage type” group was 91%, and the follow-up rate of the “reverse procedure” group was 88.8% within 3 months. There were studies show that the incidence of postoperative complications in the laparoscopic-assisted total gastrectomy for gastric cancer ranged between 10 and 40% [31, 32], and Our research results are also in this range. There was no significant difference in overall complications between the two groups, these scores were comparable to rates reported in other studies [31]. Unfortunately, 2 cases of duodenal stump leakage occurred in the cabbage type group after surgery, and 1 case of anastomotic leakage occurred in the reverse procedure group. All patients were improved and discharged after treatment. The incidence of “reverse procedure” group was lower than the “cabbage type” group in terms of recurrence and metastasis; but there was no statistical significance (*P* = 0.093) between the two groups. Nevertheless, we think the difference will be statistically significant, with number of cases increasing. One case of death due to tumor progression occurred in the two groups, respectively, which was not statistically significant. It further verified the safety and efficacy of reverse rolling-mat type lymph node dissection.

Our study has some limitations. First, this was a retrospective single-center study with a relatively small sample size. Second, although the survival rate of “reverse procedure” seem to be no less than that of “cabbage type” group, we lack more convincing evidence of 5-year survival. From the perspective of evaluation of the safety and feasibility of surgery, this will not have much impact on the study. Further large-scale survival analysis will help to verify this result, and it is also the direction that we will study next.

Overall, both the two different pattern of lymph node dissection can reach the standard of D2 lymph node dissection for radical gastric cancer, and there are no significant differences in terms of hospitalization time, incision length, postoperative complications, and long-term prognosis between the two groups. On the premise of retaining the tactile feedback and flexibility of the hands in hand-assisted radical gastric cancer surgery, we have completely solved the difficulties of laparoscopic lymph node dissection by optimizing the method of lymph node dissection. Our results suggest that the novel method in addition to optimizing surgical procedures, it can also reduce intraoperative bleeding obviously. Although the increase in the number of lymph nodes is partly due to the results of the clinicians and pathologists examined corporately, it is undeniable that changes of the surgical procedures played an important role.

## Conclusion

The pattern of reverse rolling-mat type lymph node dissection in HALTG perform well in terms of safety and feasibility. The reverse procedure makes the surgical procedure more completely and smoothly, and that meets the principle of tumor-free surgery more. The optimized approach was systematically articulated that can provide surgeons with a new perspective to solve the problem of lymph node dissection in gastric cancer surgery.

## Data Availability

The datasets used and analysed during the current study available from the corresponding author on reasonable request.

## Abbreviations

HALG: Hand-assisted laparoscopic D2 radical gastrectomy
HALTG: Hand-assisted laparoscopic D2 radical total gastrectomy
LAG: Laparoscopic-assisted radical gastrectomy
BMI: Body mass index
